# Antidiabetic Effect of Tibetan Medicine Tang-Kang-Fu-San in db/db Mice via Activation of PI3K/Akt and AMPK Pathways

**DOI:** 10.3389/fphar.2017.00535

**Published:** 2017-08-24

**Authors:** Bailu Duan, Zhongqiu Zhao, Weifang Liao, Hui Xiong, Sisi Liu, Liang Yin, Tiexiang Gao, Zhinan Mei

**Affiliations:** ^1^College of Basic Medicine, Hubei University of Chinese Medicine Wuhan, China; ^2^Center for the Study of Itch, Department of Anesthesiology, Washington University School of Medicine, St. Louis MO, United States; ^3^Barnes-Jewish Hospital, St. Louis MO, United States; ^4^College of Pharmaceutical Sciences, South-Central University for Nationalities Wuhan, China

**Keywords:** traditional Tibetan medicine, diabetes, Akt, AMPK, db/db mice

## Abstract

This study was to investigate the anti-diabetic effects and molecular mechanisms of Tang-Kang-Fu-San (TKFS), a traditional Tibetan medicine, in treating type 2 diabetes mellitus of spontaneous diabetic db/db mice. Firstly HPLC fingerprint analysis was performed to gain the features of the chemical compositions of TKFS. Next different doses of TKFS (0.5 g/kg, 1.0 g/kg, and 2.0 g/kg) were administrated via oral gavage to db/db mice and their controls for 4 weeks. TKFS significantly lowered hyperglycemia and ameliorated insulin resistance (IR) in db/db mice, indicated by results from multiple tests, including fasting blood glucose test, intraperitoneal insulin and glucose tolerance tests, fasting serum insulin levels and homeostasis model assessment of IR analysis as well as histology of pancreas islets. TKFS also decreased concentrations of serum triglyceride, total and low-density lipoprotein cholesterol, even though it did not change the mouse body weights. Results from western blot and immunohistochemistry analysis indicated that TKFS reversed the down-regulation of p-Akt and p-AMPK, and increased the translocation of Glucose transporter type 4 in skeletal muscles of db/db mice. In all, TKFS had promising benefits in maintaining the glucose homeostasis and reducing IR. The underlying molecular mechanisms are related to promote Akt and AMPK activation and Glucose transporter type 4 translocation in skeletal muscles. Our work showed that multicomponent Tibetan medicine TKFS acted synergistically on multiple molecular targets and signaling pathways to treat type 2 diabetes mellitus.

## Introduction

Diabetes mellitus (DM) is a well-known group of metabolic disorders characterized by hyperglycemia being resulted from abnormal insulin secretion and/or action (Rochester and Akiyode, 2014). DM affects millions of people worldwide, with a rapidly increasing incidence and prevalence. According to the International Diabetes Federation’s statistics in 2013, 382 million people worldwide have diabetes, more than 90% of them have type 2 diabetes (T2DM), and even this number will increase to 592 million by 2035 (Rochester and Akiyode, 2014; Saisho, 2015). Insulin resistance (IR) occurs when the insulin-sensitive tissues (mainly skeletal muscle, adipose tissue, and liver) lose their ability to respond properly to insulin (Hirabara et al., 2012), which has been assumed as a major pathophysiological feature of T2DM (Brunetti et al., 2014; Saisho, 2015). Due to its numerosity and long-term complications, such as nephropathy, retinopathy, neuropathy, and cardiovascular disorders, etc., optimal treatments and prevention strategies for T2DM are urgently needed (Rochester and Akiyode, 2014; Saisho, 2015; Tangvarasittichai, 2015).

Clinically there are several available oral agent classes, including sulfonylureas, meglitinides, biguanide, α-glucosidase inhibitors, dipeptidyl peptidase-4 inhibitors, dopamine agonists, bile acid sequestrants, thiazolidinediones and/or their combinations (Rochester and Akiyode, 2014; Marin-Penalver et al., 2016). Unfortunately due to their cost, adverse effects, etc., and most importantly their narrow targeting spectrum, it becomes increasingly needed to develop multi-targeting drugs to treat diseases by involving multiple factors and pathways (Tian and Liu, 2012; Rochester and Akiyode, 2014; Marin-Penalver et al., 2016). Herbal medicines have been used widely, especially in developing countries, to treat the T2DM (Xiao and Högger, 2015; Loizzo et al., 2016; Vinayagam et al., 2017). Accumulating experimental data and clinical trials supported that herbs, as multi-component complex interacting with multiple targets and functions, have their unique advantages in treating complex chronic diseases afflicting modern populations, like T2DM, but cause less drug resistance (Tian and Liu, 2012; Rochester and Akiyode, 2014; Marin-Penalver et al., 2016).

Tang-Kang-Fu-San (TKFS), a traditional Tibetan medicine developed with herbal formula strictly following the principles in “rGyud-bzhi”(a principal textbook of Tibetan medicine), has been widely used to treat T2DM for many years in China, especially in the Qinghai-Tibet Plateau. TKFS consists of 11 medicinal herbs including *Berberis Kansuensis Schneid*, *Curcuma longa L*, *Phyllanthus emblica* etc., which has been reported to be clinically effective, however, scientific evidence for the efficacy and exact mechanisms for the anti-diabetic activities of TKFS are still lacking. Therefore, in order to obtain more experimental evidence for a better clinical use of TKFS, the present study analyzed the features of its chemical compositions, studied the anti-diabetic effects and the possible intracellular mechanisms of TKFS in the db/db mice, a spontaneous T2DM animal model (Wang et al., 2014).

## Materials and Methods

### HPLC Fingerprint Analysis

The herbal formula of TKFS, was provided by Tibet Autonomous Region Institute of Traditional Tibetan Hospital. For each batch, TKFS (0.5 g) were accurately weighed, and extracted with 50 mL of 50% methanol in an ultrasonic water bath for 20 min. Additional 50% methanol was added to adjust the volume, and then the solvent was filtered through a 0.22 μm microfiltration membrane.

The multiple-components of the TFKS were analyzed with the Agilent 1260 HPLC system (Karlsruhe, Germany), including a quaternary solvent delivery system, an on-line degasser, an auto-sampler, a column temperature controller and a photodiode array detector coupled with an analytical workstation. The samples were analyzed using a Waters SunFire C18 column (250 mm × 4.6 mm, 5 μm) at 30°C. The binary gradient elution system consisted of methanol (A) and 0.2% phosphoric acid (B), and separation was achieved using the following gradient program: 0–15 min, 97% B; 15–16 min, 97–85% B; 16–90 min, 85–50% B. The flow rate was set at 1.0 mL/min and the sample injection volume was 10 μL. The detection wavelength was set at 273 nm.

Data analysis was performed by a professional software named Similarity Evaluation System for Chromatographic Fingerprint of Traditional Chinese Medicine composed by Chinese Pharmacopoeia Committee (Version 2009 A), which was recommended by China Food and Drug Administration (CFDA) (Tang et al., 2014; He et al., 2015).

### Animals and Drugs

Seven-week-old male diabetes spontaneous diabetic mutation *Lepr^db^* mice (referred as db/db mice, *n* = 30, body weight, 39.5 ± 1.6 g) and non-diabetic wild type littermates (referred as WT mice, *n* = 6, body weight, 19.9 ± 0.8 g) were purchased from the Model Animal Research Center of Nanjing University (Nanjing, China). They were housed in SPF animal rooms at a 12 h light–dark cycle with the suitable relative humidity (55 ± 5%) and temperature (22 ± 2°C). Before the experiment all mice were acclimated for 1 week to the environment. All experimental animal procedures followed international guidelines for care and use of laboratory animals and were approved by the Animal Ethics Committee of South-central University For Nationalities. Metformin tablets were purchased from Beijing Jing Feng Pharmaceutical Factory (Beijing, China).

### Experimental Process

The db/db mice were randomly divided into five groups (*n* = 6 per group): db/db mice plus 0.9% saline group (model control group), db/db mice plus low dose of TKFS group (TKFS 0.5 g/kg), middle dose of TKFS group (TKFS 1.0 g/kg), and high dose of TKFS group (TKFS 2.0 g/kg), db/db mice plus metformin group (Metformin 200 mg/kg), and WT mice plus 0.9% saline group (*n* = 6 as normal control group). According to the directions on the label that have used in human beings (6 g/day per adult), dose conversion from human to mice was converted according to body surface area and eventually the doses of TKFS were decided above for the mice.

Both TKFS and metformin were dissolved in 0.9% saline, and mice were treated by oral gavage administration with doses described above once a day for 4 weeks. At the end of the last week, all mice were fasted overnight. After testing FBG, all mice were sacrificed with an overdose of intraperitoneal injection of pentobarbital (90 mg/kg). The mouse blood will be quickly collected and immediately centrifuged (4°C, 300 g for 10 min) to gather the blood serum samples, which were subsequently stored at -20°C for further analysis. The pancreas tissues were dissected and immediately immersed in 4% paraformaldehyde for histological analysis. The skeletal muscles were dissected and immediately frozen in liquid nitrogen or fixed in 4% paraformaldehyde for western blot or immunohistochemical analysis.

### Fasting Blood Glucose (FBG) and Body Weight

Fasting blood glucose and body weight were measured at 08:00 to 08:30 on the first day of each week during the treatment after fasted overnight. Blood samples were obtained by tail-prick and measured by using a standard glucometer (LifeScan, Inc., Milpitas, CA, United States). The body weight was measured with an electronic weighing scale.

### Intraperitoneal (i.p.) Insulin Tolerance Test (IPITT) and i.p. Glucose Tolerance Test (IPGTT)

In the IPITT test, after the mice were fasted for 6 h at the second week of the experiment, they were i.p. injected with insulin at 0.75 IU/kg as reported previously (Liu et al., 2014; Zhang et al., 2014), and blood glucose levels were monitored at 0, 30, 60, and 120 min after the insulin injection. The total area-under-the-curve (AUC) of glucose from the sampling period from 0 to 120 min was determined as the AUC value (Runtuwene et al., 2016).

The IPGTT was performed on mice at third week of treatment with TKFS after fasted overnight, then D-glucose (0.75 g/kg) was carried out by i.p. injection to each mice (Zhang et al., 2014). The blood glucose levels as well as AUC values were recorded as described above.

### Fasting Serum Insulin Levels (FINS) and Homeostasis Model Assessment of Insulin Resistance (HOMA-IR) Analysis

Fasting serum insulin level were measured by using an enzyme-linked immunosorbent assay mouse insulin ELISA kit (CSB-E05071m, CUSABIO BIOTECH CO., Ltd, Wuhan, China). IR value was calculated according to a previous study (Dong et al., 2015), by using a formula as following: HOMA-IR = FINS (μU/mL) × FBG (mmol/L) /22.5.

### Biochemical Analysis

The serum total cholesterol (TC), triglycerides (TG), and low-density lipoprotein cholesterol (LDL-C) levels were detected by using enzymatic colorimetric kits and following the product instructions (Nanjing Jiancheng Bioengineering Institute, Nanjing, China).

### Histological Analysis of Pancreas Tissues

The tails of pancreas from each mouse were fixed in 4% paraformaldehyde, followed by processing of paraffin embedding, tissue sectioning, and hematoxylin and eosin (HE) staining. Morphological structure of islet of pancreas was observed and photographed by using an optical microscope (BH-2, Olympus, Japan).

### Immunohistochemical Analysis

The paraffin sections were deparaffinized in xylene and rehydrated through graded washes of ethanol as described previously (Ma et al., 2015). After antigen retrieval with high-temperature heating in a citrate buffer, the slides were incubated with 3% H_2_O_2_ buffer for 10 min and washed it out with PBST, then incubated with anti-GLUT4 (ab33780, Abcam, Cambridge, United Kingdom) at 1:100 dilution at 4°C overnight. After washed with PBST, the slides were incubated with biotin-labeled secondary antibody (PV-6001 Zhongshan Jinqiao Co., Ltd, Beijing, China) at room temperature for 2 h. Then the sections were incubated with avidin-biotin-peroxidase complex (1:50, Elite ABC Ki, Vector) at room temperature for 2 h. Finally after washed with PBST, the sections were incubated with a reaction buffer containing 0.02% (w/v) DAB and 0.003% (v/v) H_2_O_2_ in 0.01 M Tris-HCl (pH 7.4) at room temperature for 5–10 min. After DAB staining, the slides were washed, dehydrated again and mounted, finally observed and photographed under an optical microscope (BH-2, Olympus, Japan).

### Western Blot Analysis

The total protein from the skeletal muscles were extracted as described previously (Yang et al., 2015). Next the protein was fractionated on 10% sodium dodecyl sulfate-polyacrylamide gel electrophoresis (SDS-PAGE) and transferred onto PVDF membrane. After blocking with 5% non-fat milk for 1 h, the membranes were incubated with anti-Akt (#4685), anti-phospho-AktSer473 (#4060), anti-GLUT4 (#2213), anti-AMPKα (#5831), phospho-AMPKα (Thr172) (#2535) (CST, Danvers, MA, United States) and anti-GAPDH (Proteintech, Wuhan, China) primary antibodies at 1:1000 dilution at 4°C overnight. Then the membrane were washed in TBST and incubated with appropriate horseradish peroxidase-conjugated secondary antibodies. The protein bands were visualized by using a BeyoECL Plus (P0018, Beyotime Biotechnology, China), and a densitometry analysis was performed by using ImageJ software (NIH, Bethesda, MD, United States). GAPDH was used as the internal control for semi-quantitative analysis.

### Statistical Analysis

All data were expressed as the means ± SEM. GraphPad Prism 5 software (San Diego, CA, United States) was used for the data statistical analysis and graphics. Unpaired *t*-test was used to analyze statistical comparisons between two groups. Multiple comparisons were compared by one-way analysis of variance (ANOVA) followed by Bonferroni’s *post hoc* tests. *p-*value < 0.05 was assumed as statistically significant.

## Results

### HPLC Fingerprint Analysis of TKFS

**FIGURE 1:**
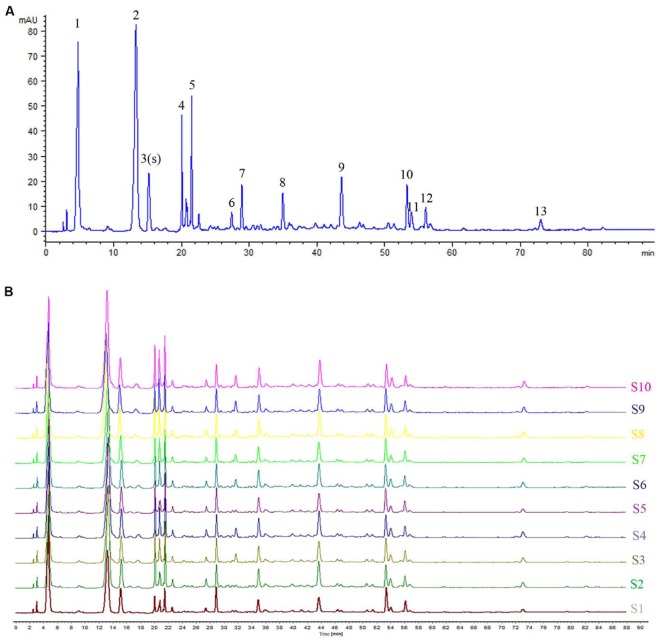
HPLC fingerprint analysis of TKFS. The HPLC chromatographic of TFKS at 273 nm wavelength. In total 13 peaks were labeled, the peak 3 is Gallic acid and the peak 13 is Curcumin **(A)**. HPLC fingerprint of 10 batches of TKFS was also presented **(B)**.

Based on the results of determination, HPLC fingerprints for TKFS were established. Reference chromatographic fingerprint for TKFS was generated based on 10 samples. A good separation and reproducible chromatogram was achieved and 13 peaks were marked as the common peaks (from peak 1 to peak 13) (**Figure 1A**) in the chromatograms of the 10 batches (**Figure 1B**). Our developed method successfully determined their features, especially those identities of Gallic acid (the 3rd peak of TKFS sample at 273 nm in **Figure 1A**), the most important active constituent of TKFS, was verified and chosen (showed by Peak 3) to calculate the relative retention time (RRT) and relative peak area (RPA) of all the other peaks (**Table 1**). The results from the 10 batches of samples (**Figure 1B**) indicated that the RPAs of the 13 common peaks (**Figure 1A**) varied, but the RRT was invariable for the herb. The similarities of the TKFS were calculated, compared with the reference chromatogram, the least similarity value of these samples was 0.90, which indicated that the samples had similar chemical compositions and this reference chromatogram could be applied as a standard HPLC fingerprint.

**Table 1:** Relative peak area.

No.	1	2	3(s)	4	5	6	7	8	9	10	11	12	13
S1	4.05	3.71	1.00	0.32	0.47	0.20	0.70	0.46	0.64	0.83	0.21	0.38	0.15
S2	3.38	5.30	1.00	0.78	1.05	0.33	0.57	0.58	1.08	0.65	0.33	0.33	0.22
S3	3.33	5.09	1.00	0.54	0.76	0.26	0.53	0.58	1.09	0.63	0.38	0.31	0.27
S4	3.57	4.74	1.00	0.46	0.69	0.26	0.59	0.59	0.99	0.71	0.35	0.46	0.24
S5	3.32	5.16	1.00	0.66	0.93	0.30	0.54	0.58	1.09	0.63	0.35	0.32	0.24
S6	3.78	4.42	1.00	0.52	0.71	0.24	0.63	0.49	0.76	0.73	0.25	0.36	0.18
S7	3.46	4.84	1.00	0.54	0.73	0.28	0.58	0.31	0.99	0.69	0.32	0.34	0.22
S8	3.62	4.40	1.00	0.41	0.58	0.24	0.63	0.56	0.90	0.75	0.30	0.36	0.20
S9	3.43	5.20	1.00	0.60	0.79	0.28	0.55	0.61	1.07	0.64	0.37	0.33	0.25
S10	3.41	5.23	1.00	0.67	0.89	0.31	0.55	0.60	1.08	0.65	0.36	0.34	0.24


### The Effect of TKFS on FBG and Body Weights

**FIGURE 2:**
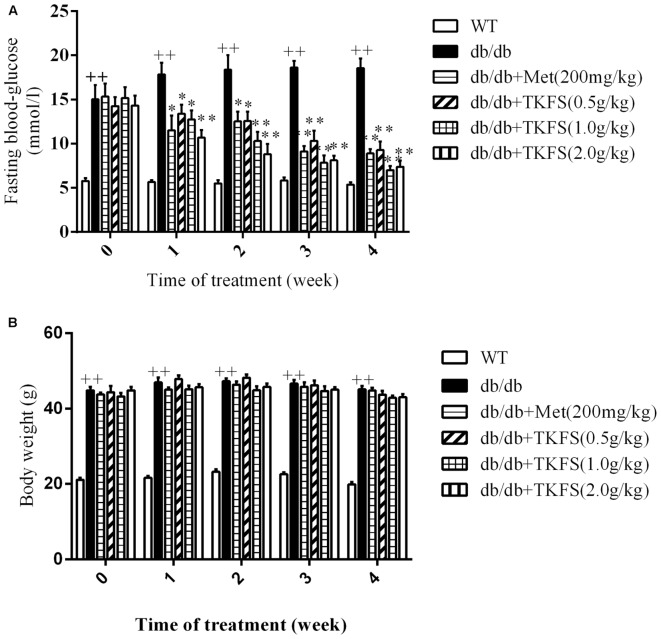
Effects of TKFS on fasting blood glucose (FBG) **(A)** and body weight **(B)**. FBG and body weight were measured at 08:00 to 08:30 on the first day of each week. ^++^*p* < 0.01 vs. WT; ^∗^*p* < 0.05, ^∗∗^*p* < 0.01 vs. db/db. Results are presented as means ± SEM and *n* = 6 in each group.

The FBG levels and body weights in model control group were significantly increased than those in WT group in all weeks we studied (*p* < 0.01) (**Figures 2A,B**). Compared to the model control, all groups with TKFS at 0.5 g/kg, 1.0 g/kg and 2.0 g/kg as well as metformin significantly lowered FBG levels than those in model control group (*p* < 0.05 or *p* < 0.01) (**Figure 2A**). However, there was no significant difference in the body weights between all db/db groups treated with TKFS at different doses or metformin to that with saline (*p* > 0.05) (**Figure 2B**).

### The Effect of TKFS on Glucose Tolerance and Insulin Tolerance

**FIGURE 3:**
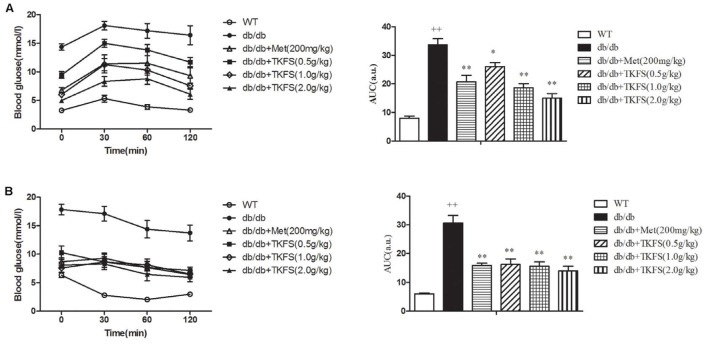
Effects of TKFS on intraperitoneal (i.p.) glucose tolerance test (IPGTT) and i.p. insulin tolerance test (IPITT). Intraperitoneally (i.p.) injected D-glucose for IPGTT **(A)**, or insulin for IPITT **(B)** were measured in each group after treatment and their total area-under-the-curve (AUC) values were determined respectively. ^++^*p* < 0.01 vs. WT; ^∗^*p* < 0.05, ^∗∗^*p* < 0.01 vs. db/db. Results are presented as means ± SEM (*n* = 6 each group).

In IPGTT and IPITT test, the TKFS (0.5 g/kg, 1.0 g/kg, and 2.0 g/kg) and metformin obviously lowered the levels of AUC in db/db mice (showed that in model control group), which are significantly increased than those in WT group (normal control group) (*p* < 0.05, *p* < 0.01) (**Figures 3A,B**).

### The Effect of TKFS on HOMA-IR Index

**FIGURE 4:**
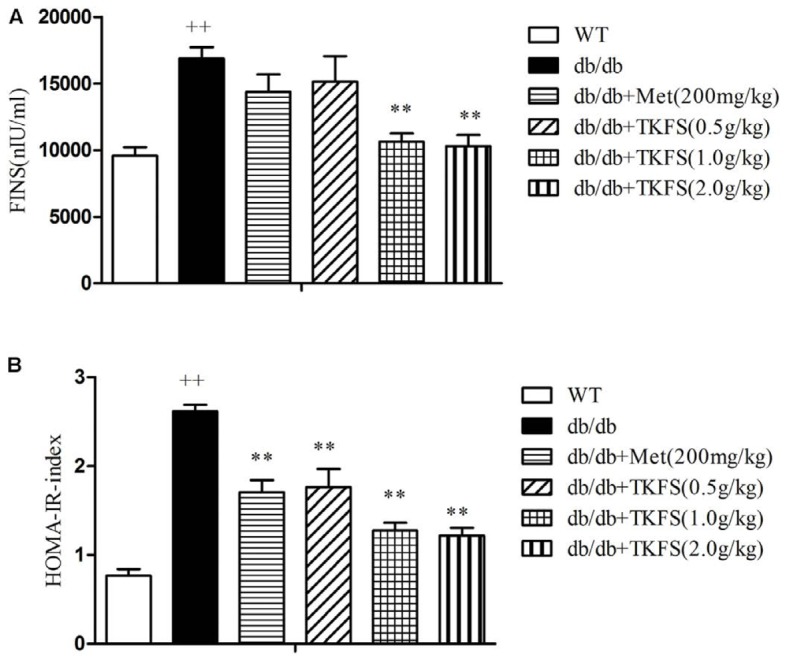
Effects of TKFS on fasting serum insulin levels (FINS) and homeostasis model assessment of insulin resistance (HOMA-IR). FINS **(A)** and HOMA-IR index **(B)** were detected in each group after 4 weeks of treatment. ^++^*p* < 0.01 vs. WT; ^∗∗^*p* < 0.01 vs. db/db. Results are presented as means ± SEM (*n* = 6 each group).

The levels of FINS and the HOMA-IR index were markedly enhanced in db/db diabetic mice compared to those in WT mice. TKFS (1.0 g/kg and 2.0 g/kg) significantly decreased the insulin level compared to the model control group (*p* < 0.01), and the HOMA-IR was significantly reduced as well (*p* < 0.01) (**Figures 4A,B**). There were no detectable changes in the FINS of db/db mice treated with TKFS (0.5 g/kg) and metformin compared with their vehicle controls (**Figure 4A**), but both of them, decreased the HOMA-IR index significantly (**Figure 4B**).

### The Effect of TKFS on Serum Biochemistry

**FIGURE 5:**
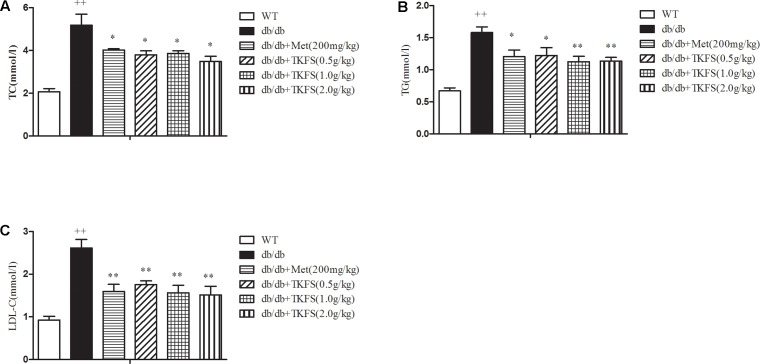
Effects of TKFS on the **(A)** total cholesterol (TC), **(B)** triglycerides (TG), and **(C)** low-density lipoprotein cholesterol (LDL-C) levels. The total TC, TG, and LDL levels in mouse blood serum were measured in each group after 4 weeks of treatment. ^++^*p* < 0.01 vs. WT; ^∗∗^*p* < 0.05, ^∗∗^*p* < 0.01 vs. db/db. Results are presented as means ± SEM (*n* = 6 each group).

The serum concentrations of TC, TG and LDL-C in the model control group were markedly increased than those in the WT group (*p* < 0.01). TKFS (1.0 g/kg and 2.0 g/kg) and metformin treatments all significantly inhibited the elevation in the levels of serum TC, TG, LDL-C of the model control db/db mice group (*p* < 0.05, *p* < 0.01) as shown in **Figures 5A–C**.

### Effects of TKFS on the Pathomorphism of Pancreas Tissues

**FIGURE 6:**
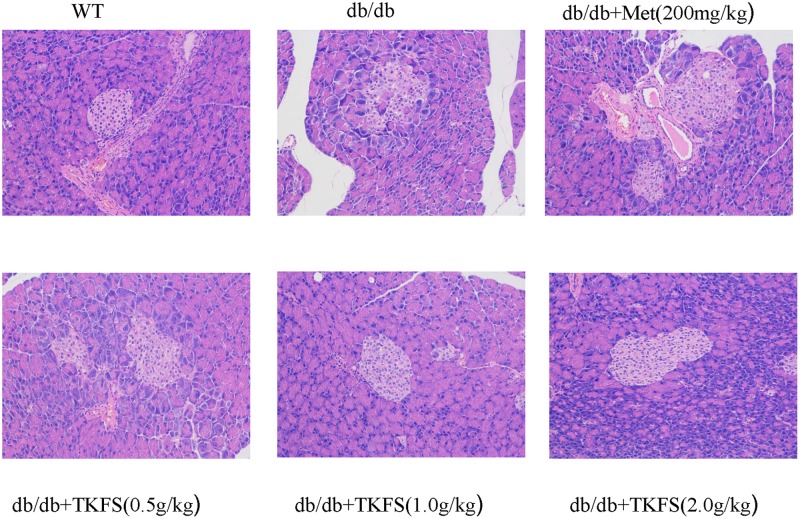
Effects of TKFS on the pathomorphism of pancreas tissues. Representative pictures of hematoxylin and eosin (HE) staining of the tails of pancreas from each group mice after 4 weeks of treatment. (×200, *n* = 3 each group).

Reduced or impaired β-cell function is one of the typical components in the pathogenesis of T2DM and previous studies clearly supported that it is essential to preserve or rescue the β-cell function as treating the T2DM (Saisho, 2015). As shown in **Figure 6**, compared to the WT group, the model control group db/db mice showed more frequently pathological changes, such as: atrophy of islets, islet cells necrosis, lacking organization of islet cells, vanished pancreas and pancreatic acini boundaries, or hypertrophy islet cells, etc. These abnormal histological changes were significantly alleviated in the TKFS (1.0 g/kg and 2.0 g/kg) and metformin treatment group compare to the model control group (**Figure 6**).

### The Effect of TKFS on p-Akt, p-AMPK and GLUT4 Expression in Skeletal Muscle

**FIGURE 7:**
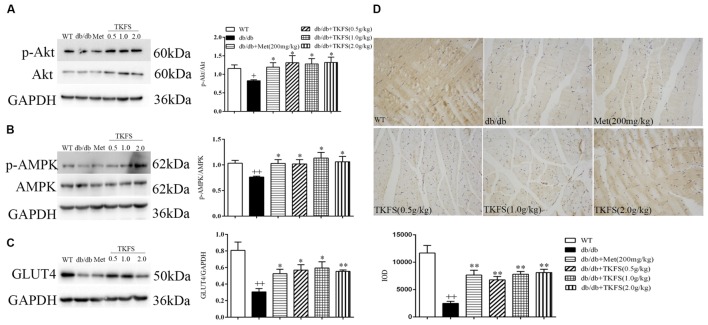
Effects of TKFS on p-Akt, p-AMPK and GLUT4 expression in skeletal muscles by western blot **(A–C)** analysis or immunochemistry **(D)**. All mice in each group mice were after 4 weeks of treatment. ^++^*p* < 0.01 vs. WT; ^∗^*p* < 0.05, ^∗∗^*p* < 0.01 vs. db/db. Results are presented as means ± SEM (*n* = 3 each group).

To evaluate whether TKFS regulates blood glucose and ameliorates IR via PI3K/Akt or AMPK signaling pathways, levels of p-Akt and p-AMPK in skeletal muscles were determined by western blot (**Figures 7A,B**), and GLUT4 were determined by western blot (**Figure 7C**) and immunochemistry analysis (**Figure 7D**) respectively. As shown in **Figures 7A,B**, the p-Akt and p-AMPK levels were significantly reduced in model control group compared with that in WT group (*p* < 0.01). TKFS (0.5 g/kg, 1.0 g/kg, and 2.0 g/kg) and metformin significantly reversed the down-regulation of p-Akt and p-AMPK expression compared with that in model control group (*p* < 0.05). The GLUT4 protein expression was dramatically lower in model control group than that in WT group (*p* < 0.01). Treatment with TKFS (0.5 g/kg, 1.0 g/kg, and 2.0 g/kg) or metformin significantly normalized the protein expression of GLUT4 in skeletal muscles down-regulated in the model control group (**Figures 7C,D**).

## Discussion

It is well-known that over 5,000 years traditional Chinese medicine had used numerous herbal formulas to form multi-ingredient herbal medicine applied to various types of diseases. T2DM is the most common form of DM in worldwide diabetic patients, which mostly exhibit obesity, hyperglycemia and dyslipidemia (high triglyceride and low high-density lipoprotein cholesterol levels, postprandial hyperlipemia) (Triplitt et al., 2000; Rochester and Akiyode, 2014). Due to the complexity of T2DM pathogenesis and severe secondary complications in multiple systems, it is much difficult to expect a single available medicine to prevent or treat all the events. Meanwhile, those herbal medicines or nutritional therapies to multiple targets may potentially prevent and control this metabolic disease after their effectiveness and interacting mechanisms are clearly elucidated (Xiao and Högger, 2015; Loizzo et al., 2016; Vinayagam et al., 2017).

With this idea, the present study focused on studying the therapeutic efficacy of a traditional Tibetan medicine, TFKS, to treat T2DM. The HPLC analysis was used to characterize the phytochemical features of this multi-herbal formulae. The 13 common peaks (shown in **Figure 1**) were proposed as the fingerprints of multiple chemical constituents of TKFS. Among them the peak 3 was identified as Gallic acid, which has been reported as a health food ingredient preventing DM (Huang et al., 2016), and showed more promising effect than resveratrol and metformin in decreasing oxidative stress-related diabetic complications (Kaur et al., 2016). Furthermore, clinical trials showed another constituent in TKFS is Curcumin, which was identified at 430 nm (data not shown) has significant antioxidant effects (Panahi et al., 2017), or treats hyperglycemia and ameliorates dyslipidemia effectively (Neerati et al., 2014; Mirzaei et al., 2017) in patients with T2DM. These recent studies were encouragingly reinforced by the results from TFKS. However, more scientific information, such as the exact component and what potential role of each component playing to treat T2DM in TFKS, still require us to conduct more studies.

Leptin receptor-mutant (db/db) mice were reported as a popular animal model of T2DM (Wang et al., 2014), with syndromes including hyperglycemia, obese and hyperlipidemia, etc. (Islam, 2013; Wang et al., 2014). The pathogenesis and process of T2DM in db/db mice perfectly resembled those in human DM diseases (Wang et al., 2014). To study anti-diabetic effects of TKFS in treating T2DM, 8-week-old db/db mice were used in our work. As known in previous studies or claimed by the providers (Triplitt et al., 2000; Islam, 2013), the db/db mice were diabetic and obese during the entire experiment compared with the WT group mice. Consistently, all db/db mice used in this study had developed a stably higher FBG than that in WT mice, and TKFS effectively decreased blood glucose in all the 4 weeks detected (shown in **Figure 2**). However, even though TKFS was able to ameliorate dyslipidemia significantly (shown in **Figure 5**) but not affected the body weight much (shown in **Figure 2**) in db/db mice. Because of the complexity of pathogenesis of T2DM, a relative large proportion of T2DM patients are not obese at all. Therefore, the “neutral” effect on body weight of TKFS, even though a bit surprising, would suggest TKFS as a suitable treatment for the population of not obese T2DM patients.

Also it is well-known that T2DM disorders typically associated with impaired glucose tolerance and insulin resistance (Triplitt et al., 2000). Reduced sensitivity to insulin in peripheral target tissues such as liver, muscle, and adipose tissue leading to abnormal insulin secretion, ultimately result in hyperglycemia (Abdulghani, 2013; Cornell, 2015). Insulin resistance in the peripheral target tissues, particularly in skeletal muscles, is considered the major cause and main therapeutic target of insulin resistance in T2DM (Stanford and Goodyear, 2014; Cornell, 2015). In the present study, the i.p. glucose tolerance test and i.p. insulin tolerance test were detected (shown in **Figure 3**), which clearly indicated that a weakened glucose tolerance and an impaired insulin tolerance were observed in the db/db mice. TKFS improved glucose tolerance and against insulin resistance, (shown in **Figures 3**, **4**), and alleviated the pathological changes in pancreas tissues (shown in **Figure 6**), which further interpreted how TKFS possibly achieves its promising therapeutic effects to treat T2DM.

There are at least two pathways known as PI3K and AMPK signal transduction pathways involving in the glucose metabolism (Nandipati et al., 2017). Intracellular PI3K/Akt pathway and GLUT4 are very important for the insulin-stimulated glucose intake in muscles (Wang et al., 2016; Yu et al., 2017). The activation of Akt could excite expression and translocation of GLUT4 resulting in enhanced glucose uptake and utilization (KorkmazIcöz et al., 2016; Li et al., 2017). AMPK, a serine/threonine protein kinase, is an evolutionarily conserved guardian of cellular metabolism and energy balance (Hardie, 2013), which plays a major role in maintaining glucose and lipid metabolism (Qiang et al., 2015; Nandipati et al., 2017). A lot of studies showing AMPK pathway, as a major regulatory pathway of GLUT4 translocation, can increases glucose uptake in both skeletal muscles and adipose tissue, thus contributing to improved blood glucose homeostasis (Yang et al., 2014; Seo et al., 2015; Mccarthy et al., 2016).

To verify whether TKFS also works through PI3K/Akt or AMPK pathway to play its therapeutic effect, we measured the relative protein levels in skeletal muscles by western blot or immunohistochemistry. Down-regulated p-Akt and p-AMPK in muscle tissues, as well as the reduction in GLUT4 expression in skeletal muscles in db/db mice were effectively restored by TKFS treatment (shown in **Figure 7**). In a few recent reports, both Gallic acid and Curcumin were demonstrated increasing tissue insulin sensitivity and glucose uptake, protecting pancreatic β-cells, or activating GLUT4 in through PI3K/Akt or AMPK dependent pathways (Gandhi et al., 2014; Jimenez-Flores et al., 2014). Again, the conclusion in the present study that PI3K/Akt and AMPK signaling cascades are responsible for TKFS’s anti-diabetic activities is perfectly in line with these work. Further studies are important to define the interaction other components in TKFS with Gallic acid and Curcumin, and the actual signaling pathways for them to exert their effects.

Finally whether TKFS associates with two interesting target genes for the intervention of PI3K/Akt or AMPK pathways was investigated. Peroxisome proliferator-activated receptor gamma (PPARγ) is a nuclear receptor. During inflammation and immune response, PPARγ negatively regulates the activities of other transcription factors, such as members of the NF-κB and AP-1 families (Gross et al., 2017). In tissues with high oxidative rates (skeletal muscle in the present study), PPARγ mRNA levels were significantly reduced in db/db mice compared to that in WT mice (Supplementary Figure 1A). Administration of both TKFS (1.0 g/kg and 2.0 g/kg) and metformin significantly normalized the PPARγ levels in db/db mice (Supplementary Figure 1A). NLRC3 (NLR family CARD domain containing 3), belongs to a large family of cytoplasmic sensors, regulating inflammatory and autoimmune diseases. NLRC3 suppresses PI3K/AKT pathway downstream of either receptor tyrosine kinases or Toll-like receptor 4 (TLR4) to modulate mTOR activity, thereby, preventing cellular proliferation and tumorigenesis (Karki et al., 2016, 2017). In db/db mice, the NLRC3 mRNA levels were significantly increased, but metformin and the highest TKFS (2.0 g/kg) showed potent inhibitory effect on NLRC3 expression (Supplementary Figure 1B). The data demonstrated that TKFS could associate with PPARγ and NLRC3 to regulate gene expression in inflammation, lipid and glucose metabolism, and insulin sensitization, further supporting the therapeutic interest of TKFS.

In summary, the present study demonstrated that TKFS, with its identical phytochemical features from 11 medicinal herbs, could ameliorate diabetic syndromes in db/db mice. Our data indicate that TKFS could potently improve the deficits in glucose/lipid metabolism and against insulin resistance. The underlying molecular mechanisms are at least by affecting the activity of the key signal factors in PI3K/Akt and AMPK signaling pathways directly or indirectly.

## Author Contributions

TG, ZM, ZZ, and BD conceived and designed the study. BD, ZM, and ZZ analyzed the data and wrote the manuscript. BD, WL, HX, SL, and LY performed the experiments.

## Conflict of Interest Statement

The authors declare that the research was conducted in the absence of any commercial or financial relationships that could be construed as a potential conflict of interest.
