# Supplementary material for: Antidiabetic Effect of Tibetan Medicine Tang-Kang-Fu-San in db/db Mice via Activation of PI3K/Akt and AMPK Pathways
Source: Front Pharmacol. 2017 Aug 24;8:535. doi: 10.3389/fphar.2017.00535 (PMC5573713; doi:10.3389/fphar.2017.00535)
Supplement: Supplementary file 1 [file Image_1.PDF]

Supplementary Figure 1

A

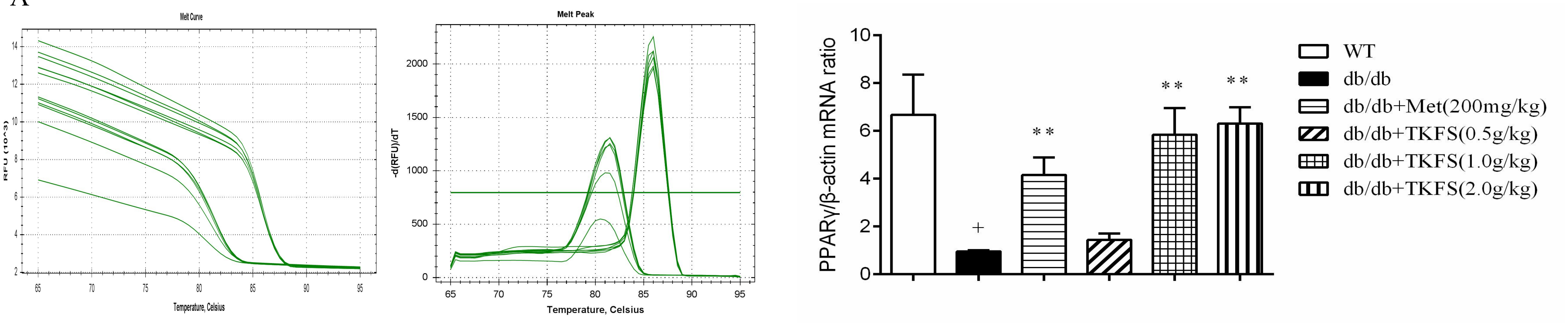

B

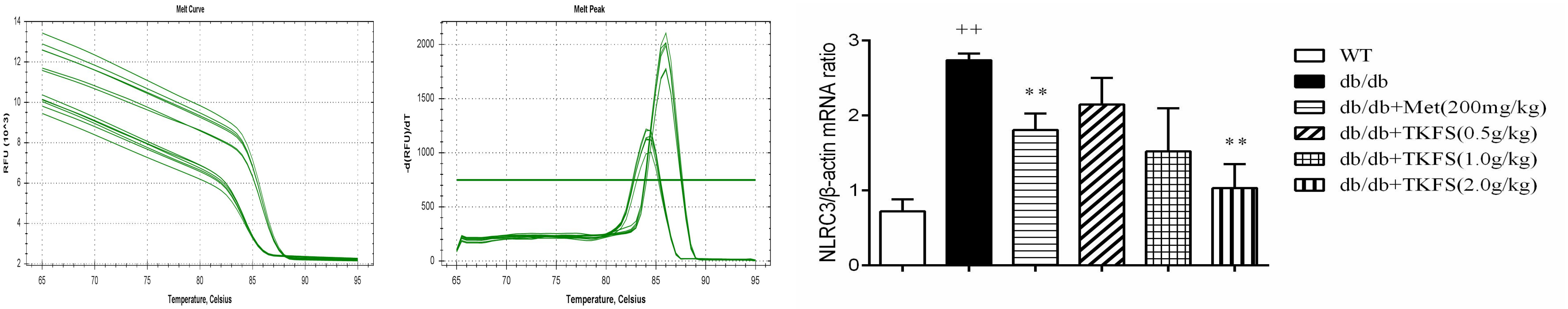

Effects of TKFS on PPARγ and NLRC3 mRNA expression in skeletal muscles. PPARγ (A) and NLRC3 (B) mRNA expression were measured by real-time PCR in each group after 4 weeks of treatment. +$p < 0.05$, ++$p < 0.01$ vs. WT; **$p < 0.01$ vs. db/db. Results are presented as means ± SEM (n = 4 each group).

The following primer sequences were employed for real-time PCR.

PPARγ 5'-GGCTGCAGCGCTAAATTCTT-3',
5'-GGAATGCGAGTGGTCTTCCA-3'

NLRC3 5'-CAGGAAGCCTGGCTGAGAAT-3',
5'-TGATCACGTTGCTTTGGAGG-3'

β-actin 5'-GTGACGTTGACATCCGTAAAGA-3',
5'-GTAACAGTCCGCCTAGAAGCAC-3'
